# In Revered Memory of Prof. N.S. Vahia, M.D

**DOI:** 10.4103/0973-1229.40572

**Published:** 2008

**Authors:** Ajai Singh

**Affiliations:** Editor, *MSM*, E-mail:mensanamonographs@hotmail.com

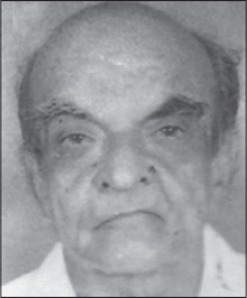



Psychiatrist, Teacher, Mentor,… Quietly Effulgent

I have to record with deep sorrow and grief the demise in June 2007 of one of the doyens of clinical psychiatry in India, Prof. N.S. Vahia.

Sharply arched eyebrows, deep-set eyes, soft spoken and gentle demeanour, excellent clinical skills, abiding interest in all branches of psychiatry, and a keen benevolent temperament. This is how most who knew, and met, Prof. N.S. Vahia will remember him.

He came on the Indian psychiatric scene and headed the Psychiatry Department at Seth G.S. Medical College and K.E.M. Hospital when psychiatry was in its infancy here. A keen clinician and researcher, he helped establish a sound clinical and research base along with his junior colleagues at this institution. His works have been published in major international journals (Vahia, 1959, 1963; Vahia *et al*., 1966, 1972, 1973, 1975). His strong commitment was to bring psychiatry into the mainstream of medicine, and to interact actively with colleagues from various other departments to remove their misconceptions and prejudices about the branch. This was carried forward by many of his illustrious successors in the department, notably Prof. V.N. Bagadia and Prof. D.N. Doongaji.

The major work for which he will be remembered in research was to understand and popularise Patanjali Yoga as a means of treatment in psychiatric disorders (Vahia *et al*., 1966, 1972, 1973, 1975). It is but fitting that a theme monograph like this, which includes well-being as one of its thrust areas, should be dedicated to someone who has done much to incorporate Patanjali Yoga into medical practice.

## Great Compassion, Sharp Intellect, and Keen Interest

However, the main reason why the vast majority of his contemporaries and juniors will remember him was the great compassion, sharp intellect and keen interest he retained right till the very end in various aspects of psychiatry. He was pleasantly approachable, and very communicative, although a man of few words. He loved writing back to those who sent him their writings, and he was quick to find merit in them, hardly ever indulging in decimating the works of others, which he could have probably done too.

It was my misfortune not to be taught by him at K.E.M., as he had retired by then. But I was fortunate to learn the ennobling and exalting aspects of this branch from every interaction I had with this benign patriarch of Indian psychiatry. His humility and patience were worthy of emulation. Just his presence in white added grace and warmth to any psychiatric function he chose to attend.

## Quiet, Selfless and Quality Work

The tradition of quiet, selfless and quality work that he has bequeathed us should stand us in good stead when noise, self-seeking and mediocrity engulf us in many spheres of life, psychiatric research included.

The torch of honest enquiry he has ignited in many who came in his wake in Indian psychiatry will also stand it in good stead as it marches forward resolutely in the 21^st^ century.

I pray the guiding principles of his life and work live on through his countless admirers, and I am fortunate to count myself as one of them. I am sure Vihang, his son and fellow psychiatrist, a keen researcher-clinician in his own right, will be proud to carry forward his legacy.

We, at *MSM*, are honoured and happy to dedicate this issue of *MSM* 2008, which is a theme issue on Medicine, Mental Health, Science, Religion and Well-being, to his loving memory.
